# Exploring the Relationship between Maternal Gatekeeping with Paternal Parenting and Adolescent Aggression

**DOI:** 10.3390/bs14070517

**Published:** 2024-06-21

**Authors:** Huaiyu Wang, Peijing Xu, Yali Jiang

**Affiliations:** 1 School of Psychology, South China Normal University, Guangzhou 510631, China; 2023023962@m.scnu.edu.cn (H.W.); 20182931020@m.scnu.edu.cn (P.X.); 2 Department of Psychology, School of Education Science, Hunan Normal University, Changsha 410081, China

**Keywords:** maternal gatekeeping behavior, paternal parenting involvement, parenting style, adolescent aggression, latent profile analysis

## Abstract

Based on the traditional Chinese cultural belief of “male breadwinner, female homemaker”, as well as the systemic and interactive characteristics of families, this study aims to explore the relationship between maternal gatekeeping behavior and the quality and quantity of paternal parenting, as well as adolescent aggressive behavior. A total of 483 seventh-grade students completed questionnaires on maternal gatekeeping behavior, paternal involvement, parenting styles, and aggressive behavior. Latent profile analysis identified four parenting combinations: positive, negative, mixed, and neglectful. Adolescents under negative parenting exhibited the highest aggression and experienced the highest maternal gatekeeping behavior, while those under positive and neglectful parenting showed the least aggression and least maternal gatekeeping behavior. Maternal gatekeeping behavior correlated with paternal negative parenting and adolescent aggression. Paternal negative parenting mediated the relationship between maternal gatekeeping and aggression, while paternal involvement moderated this relationship. These findings highlight the role of parental interaction in adolescent behavior and support family-based interventions.

## 1. Introduction

Aggression is an intentional behavior pattern that causes harm to others physically and psychologically [1]. Aggressive behaviors can take various forms, including physical punishment, bullying, discrimination, pranks, and online attacks, among others, with the extreme form being violence. According to a World Health Organization report on youth violence, over 176,000 homicides occur annually among individuals aged 15–29 worldwide, accounting for 37% of the total annual global homicide cases. Furthermore, non-lethal youth violence also has a significant impact on individuals’ physical, psychological, and social functioning, which can often be lifelong [2]. Understanding the causes and predictive factors of aggressive behavior, as well as implementing effective intervention measures, are essential in reducing teenage aggression, maintaining social safety, and promoting healthy individual development.

Teenagers’ aggressive behavior may be linked to their own psychological and emotional issues, as well as environmental and familial factors. There is evidence to suggest that family-based interventions are more effective than individual interventions [3], implicating that family plays a crucial role in teenage aggression.

### 1.1. Parenting Styles and Adolescent Aggression

Parenting style refers to a fixed pattern and tendency of behaviors parents exhibit in their daily interactions with their children, with the parent–child relationship at the center of family life. Multiple empirical studies have shown a significant association between parenting styles and teenage aggressive behavior [4,5].

There are various classifications of parenting styles. For example, Braza et al. [4] differentiated three types of parenting styles based on the interaction of parental warmth and parental control, including authoritative, authoritarian, and permissive [6]. Different parenting styles have been associated with different externalizing problems in adolescents. For instance, the authoritative parenting style (high level of parental warmth and control) has been linked to lower levels of aggression in girls, the combination of an authoritarian maternal style (low on warmth and high on control) and a permissive paternal style is positively associated with aggressive behavior in girls and boys, while higher levels of aggression have been observed in girls when parents adopt a permissive parenting style (high on warmth and low on control). Additionally, Song Minghua [1] categorized parenting styles into a positive parenting style (warmth and affection) and negative parenting style (rejection and overprotection). More importantly, their measurement incorporated the parenting styles of both fathers and mothers [7], providing more comprehensive information for understanding the relationship between parenting and adolescents’ behaviors. Based on this measurement, parental rejection emerges as a significant predictor of adolescent aggressive behavior [8,9,10]. Similarly, a one-year longitudinal study revealed that early parental rejection positively predicted later externalizing behavior in adolescents [10], aligning with previous research conducted in Asian countries [11,12,13]. However, these studies measure the overall level of parental rejection by adding up the scores from both parents; the fact that parenting style is not always additive [14], but interactive [15], between mothers and fathers has been largely neglected in these studies.

### 1.2. The Mutual Influence of Parenting between Parents

Family systems theory suggests that the marital subsystem directly affects the overall functioning and parent–child interaction within a family. Due to the traditional division of parental roles, fathers have been considered “outsiders” in child-rearing, resulting in many mothers restricting, controlling, and refusing paternal involvement in household chores and child-rearing activities [16]. These behaviors of mothers are known as gatekeeping or door-closing behavior. Mothers regulate paternal involvement in child-rearing by either facilitating or inhibiting their role in the co-parenting relationship [17,18,19], including their physical and emotional investment in terms of interaction, accessibility, and responsibility during the children’s development [20]. Rane and McBride [21] argue that the institution of marriage and the nature of the co-parenting relationship directly influence a father’s sense of identity. For example, some researchers argue that gatekeeping behavior operates indirectly through the father’s beliefs and self-efficacy in parenting, lowering their level of involvement [22]. In contrast, maternal gate opening behavior positively predicts a father’s parenting involvement [23]. Research has shown that maternal gate opening behaviors can lead to increased father involvement in various aspects, such as caregiving, creating lasting memories with the child, and engaging in play activities [24].

Maternal gatekeeping behavior not only exerts a negative impact on paternal involvement in child-rearing but also leads to a more negative parenting style in fathers, known as the maternal gatekeeping effect [25]. A pioneering study conducted by Altenburger et al. [25] explored the longitudinal connection between maternal gatekeeping behavior and the quality of fathers’ parenting and demonstrated that when confronted with maternal gatekeeping behavior, fathers’ parenting quality is negatively affected in terms of their responsiveness to infant cues, their level of active participation, and the emotional atmosphere during interactions.

Nonetheless, the majority of the existing research has primarily concentrated on the impact of maternal gatekeeping behavior on the quantity, namely, fathers’ parenting involvement [25], and less on fathers’ parenting styles. More importantly, it should be noted that the quality (paternal parenting style) and the quantity (paternal involvement) often interact with each other. In Zvara’s [26] study, it was found that in families where fathers exhibit violent tendencies, the children are more prone to behavioral problems when the fathers’ involvement was encouraged. Moreover, previous studies have predominantly focused on gatekeeping behavior and the father’s parenting involvement in infancy and early childhood, with less attention given to adolescence [25,27,28].

**Figure 1:**
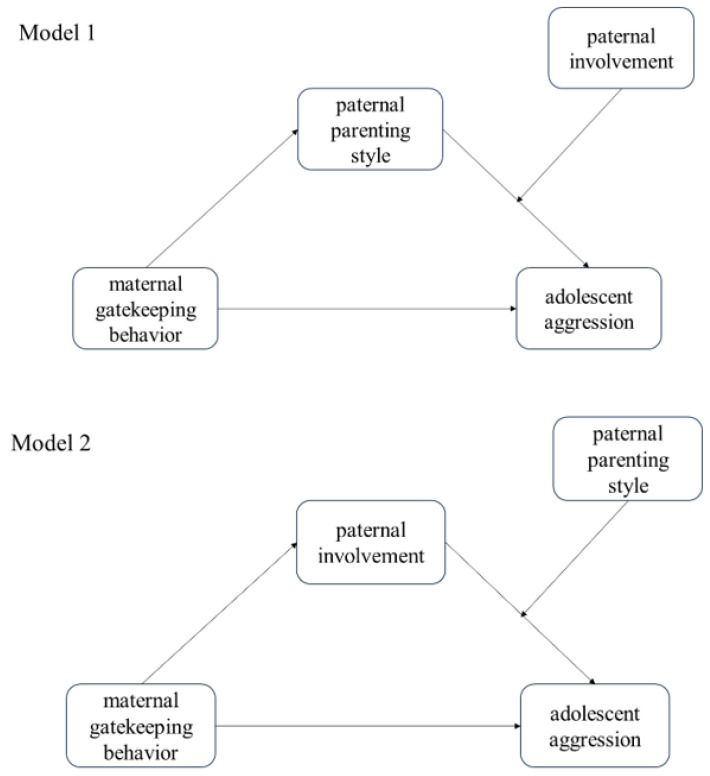
Hypotheses model.

Therefore, this study attempts to address two major questions. First, we examined the latent classes of parenting styles by incorporating both mothers’ and fathers’ parenting styles. Second, we aimed to investigate the relationship between maternal gatekeeping behavior, paternal parenting behavior (including the parenting style as the “quality” and paternal involvement in child-rearing as the “quantity”), and adolescent aggressive behavior. Based on the traditional Chinese cultural belief of “male breadwinner, female homemaker”, as well as the systemic and interactive characteristics of families, we constructed three hypotheses: (1) the mother’s gatekeeping behavior is negatively correlated with adolescent aggression; (2) the mother’s gatekeeping behavior is associated with paternal parenting behavior; (3) the father’s parenting behavior plays a mediating role between the mother’s gatekeeping behavior and adolescent aggression. Based on these three hypotheses, two models were constructed and compared (Figure 1): Model 1 considers the father’s parenting style as the mediating variable between the mother’s gatekeeping behavior and adolescent aggression, with paternal involvement in child-rearing as the moderating variable in the second half of the mediation process; Model 2 considers paternal involvement in child-rearing as the mediating variable, with the father’s parenting style as the moderating variable in the second half of the mediation process. To exclude the potential influences of family economic status and the education level of the parents based on previous evidence, we also measured these variables and controlled these influences during the model analysis [29]. By examining the relationship between maternal gatekeeping behavior and paternal parenting behavior, we can gain a better understanding of the potential dynamics within the family system and provide practical guidance for family-based therapy for adolescent aggression.

## 2. Methods

### 2.1. Subjects

A total of 527 seventh-grade students participated in this study; some were from a school in Guangzhou, Guangdong Province, and some were from another school in Haikou, Hainan Province. A total of 483 questionnaires were collected, resulting in a response rate of 91.65%. The participants had an average age of 12.66 ± 0.80 years, with 249 males and 234 females. The parental education level was coded into 3 levels, and the subjective economic status (SES) of the family was collected using a Likert scale ranging from 0 to 10 [30].

### 2.2. Self-Reported Measurements

#### 2.2.1. Maternal Gatekeeping Behavior Perception Questionnaire

The Maternal Gatekeeping Behavior Perception Questionnaire for adolescents was adapted from the Maternal Gatekeeping Behavior Questionnaire translated and developed by Zou Shengqi [15]. It consists of 11 items with two dimensions: “Perceived Maternal Gate Opening Behavior” and “Perceived Maternal Gate Closing Behavior”. The Likert scale ranges from 0 to 6, with response options “Never”, “Rarely”, “Seldom”, “Sometimes”, “Often”, “Very often”, and “Extremely often”. The Perceived Maternal Gate Closing Behavior dimension includes 7 items, measuring the adolescent’s perception of maternal refusal and the restriction of paternal involvement in parenting. Higher scores indicate a greater perception of maternal gate closing behavior. For example, an item is “My mom controls the time my dad spends with me”. The Perceived Maternal Gate Opening Behavior dimension includes 4 items, measuring the adolescent’s perception of the maternal acceptance and support for paternal involvement in parenting. For example, an item is “My mom speaks positively about my dad in front of me”. In this study, the Cronbach’s α coefficient for the Gate Closing dimension was 0.72, and that for the Gate Opening dimension was 0.78.

#### 2.2.2. Adolescent Evaluation of Parental Involvement Behavior Questionnaire

In this study, the Adolescent Evaluation of Parental Involvement Behavior Questionnaire developed by Wu Xinchun et al. [31] was used to measure the level of paternal investment in parenting. This questionnaire is completed by adolescents and is used to assess their perception of the paternal involvement in parenting. The questionnaire consists of 4 dimensions and a total of 22 items. The “Emotional Leisure” dimension includes 11 items (an example item is “Dad and I discuss the things I’m interested in”), the “Rule Teaching” dimension includes 3 items (an example item is “My father taught me that I need to be responsible for my own affairs”), the “Academic Support” dimension includes 4 items (an example item is “Dad discusses with me the difficulties I encounter in my studies”), and the “Life Care” dimension includes 4 items (an example item is “Dad takes care of my daily life and routines”). The Likert scale ranges from 0 to 4, with response options “Never”, “Occasionally”, “Sometimes”, “Often”, and “Always”. Higher scores on a particular dimension indicate that adolescents perceive higher levels of paternal investment in that dimension. In this study, the Cronbach’s α coefficients for the four dimensions ranged from 0.72 to 0.93.

#### 2.2.3. Short Form of Parental Rearing Style Questionnaire

The Short form of Parenting Rearing Style questionnaire was used in this study to measure the parenting styles of the adolescents’ parents. It was originally developed by Arrindell et al. [32] and subsequently revised by Jiang et al. [7]. The revised scale not only reduced the number of items but also ensured its cultural appropriateness. The questionnaire consists of 3 dimensions and a total of 21 items. The “Refusal” dimension includes 6 items (an example item is “Mom/Dad often yells at me for no apparent reason”), the “Emotional Warmth” dimension includes 7 items (an example item is “Mom/Dad praises me”), and the “Overprotection” dimension includes 8 items (an example item is “Mom/Dad does not let me do some things that other kids can do because she/he is afraid I will get into trouble”). The items are scored on a scale of 1 to 4, with response options “Never”, “Occasionally”, “Often”, and “Always”. Seventeen items are reverse-scored. The higher the score in a particular dimension for either the father or the mother, the more that parenting style is associated with that type. Parenting styles can differ between fathers and mothers. This study refers to the research by Song Minghua et al. [1] and explores parenting styles from the perspectives of positive parenting styles (including the Emotional Warmth dimension) and negative parenting styles (including the Refusal and Overprotection dimensions). In this study, the Cronbach’s α coefficients for the three dimensions of the father version ranged from 0.69 to 0.87, and those for the mother version ranged from 0.71 to 0.85.

#### 2.2.4. Buss and Perry Aggression Questionnaire

The Chinese version of the Buss and Perry Aggression Questionnaire, translated and revised by Li Xianyun et al. [33], was used in this study to measure the level of aggression in adolescents. The questionnaire consists of 5 dimensions and a total of 30 items. The “Physical Aggression” dimension includes 7 items (an example item is “I get into fights a bit more often than others”), the “Verbal Aggression” dimension includes 5 items (an example item is “I am prone to getting into arguments with others”), the “Anger” dimension includes 6 items (an example item is “I have difficulty controlling my temper”), the “Hostility” dimension includes 7 items (an example item is “I suspect that someone is mocking me behind my back”), and the “Self-directed Aggression” dimension includes 5 items (an example item is “When I am very frustrated, I think about hurting myself”). The items are scored on a Likert scale of 1 to 5, with response options “Not at all”, “Slightly”, “Moderately”, “Quite a bit”, and “Extremely”. Higher scores indicate higher levels of aggression in an individual. In this study, the Cronbach’s α coefficients for the five dimensions ranged from 0.71 to 0.85.

### 2.3. Procedure

In this study, the paper versions of the questionnaires were administered. To protect student privacy, the entire administration process was conducted anonymously upon the approval of them and their parents. The collected questionnaires were examined by undergraduate psychology students to remove any that did not adhere to the response guidelines or had unclear content. The data were then saved and managed in SPSS 22.0 for statistical analysis.

### 2.4. Statistical Analysis

Descriptions and correlation analyses of each variable were performed with SPSS 22.0. Mediation analyses were conducted based on the proposed Model 1 and Model 2 with SPSS PROCESS macro, with gender, SES, and education level of the parents as covariates. Mediation effects were confirmed after 5000 bias-corrected bootstrapping (*p* < 0.05).

The Latent profile analysis was conducted with M-plus 7.0. Among the six fit indices used to select the latent class model, smaller values of the Akaike Information Criterion (AIC), Bayesian Information Criterion (BIC), and Adjusted Bayesian Information Criterion (ABIC) indicate better model fit. Some scholars have suggested that the BIC is the preferred fit index among these three [34,35]. The entropy value represents the accuracy of individual classification, with higher values indicating better model fit. Generally, a value above 0.8 indicates higher classification accuracy. *p*-values of the Lo-Mendell-Rubin Likelihood Ratio (LMR LR) test and Bootstrap Likelihood Ratio Test (BLRT) less than 0.05 indicate that the k-class model is superior to the k-1 class model [36].

To better address the mutual relationship between parents and to exclude potential confounds between different types of family structures, we analyzed the whole sample, which includes conventional families (parents living together with their children and getting along well) and non-conventional families (divorce, single family, children left-behind, etc.), and the samples from conventional families only.

### 2.5. Common Method Bias

To control for common method bias, this study employed anonymous administration and reverse scoring for some items. Two different schools were involved. After collecting the data, Harman’s single-factor test was used. The results revealed 27 unrotated common factors with eigenvalues greater than 1. The largest factor accounted for 17.43% of the variance, indicating that there was no severe common method bias in this study.

## 3. Results

### 3.1. Demographics

Based on the family structure, we found differences between conventional families and non-conventional families in several variables. In general, the conventional families had lower mother gatekeeping behavior, less adolescent aggression, more positive parenting from both parents, and a higher SES compared with non-conventional families (Table 1).

### 3.2. Latent Profile Analysis

**Figure 2:**
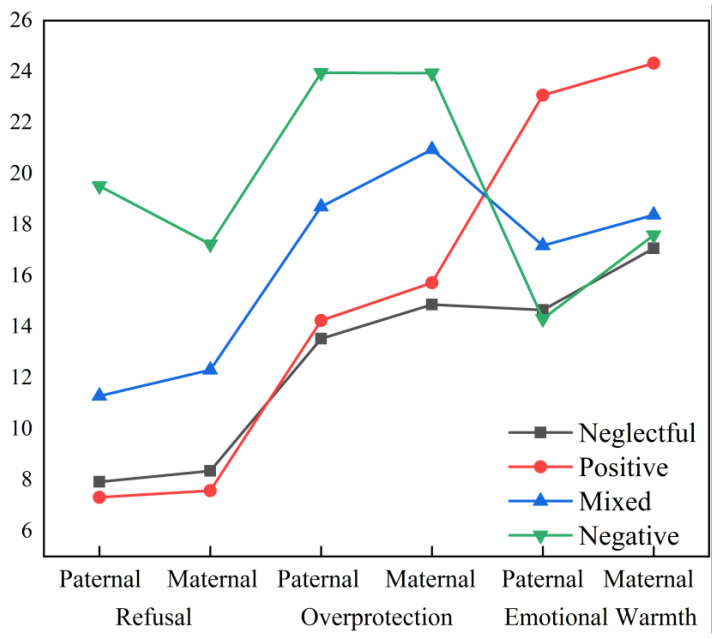
The latent classes of parental rearing styles based on LPA.

To explore the latent categories of parental rearing styles, three parenting styles (rejection, emotional warmth, overprotection) were used as observed variables. The latent profiles of parental rearing styles were analyzed using one, two, three, and four latent class models. The fit results of the analysis model are shown in Table 2. Based on the LMR LR test, the three-class model is superior to the two-class model, and the two-class model is superior to the one-class model. However, comparing the three-class model and the four-class model, it was found that the three-class model actually combines the low negative–low positive and low negative–high positive classes into one class. Taking into account the fit information mentioned above, the four-class model was ultimately chosen as the final model for latent profile analysis. The four classes are positive (positive parenting from both parents, 48.97%), negative (negative parenting from both parents, 6.82%), mixed (medium positive/negative parenting from both parents, 19.42%) and neglectful (low positive and negative parenting from both parents, 24.79%) (Figure 2).

### 3.3. Validity of the Latent Profile Analysis Results

**Figure 3:**
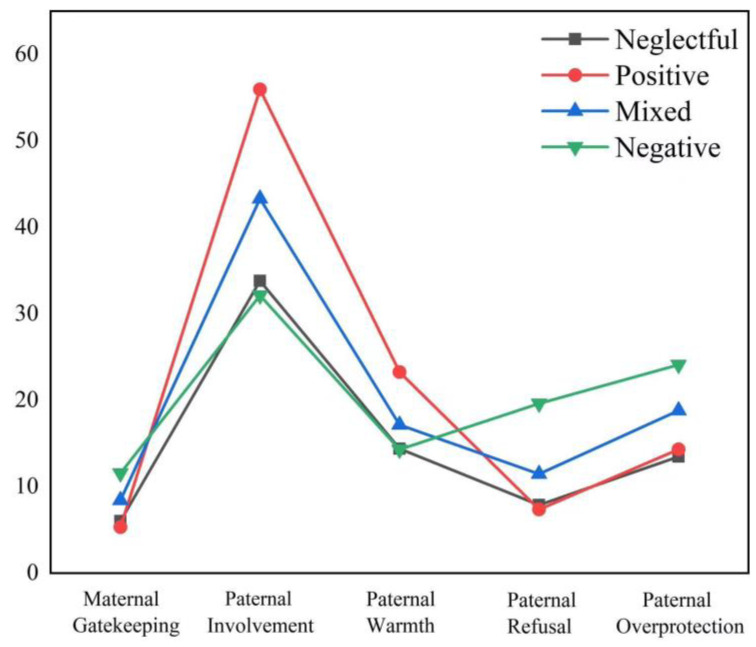
Maternal gatekeeping behavior and parental parenting.

**Figure 4:**
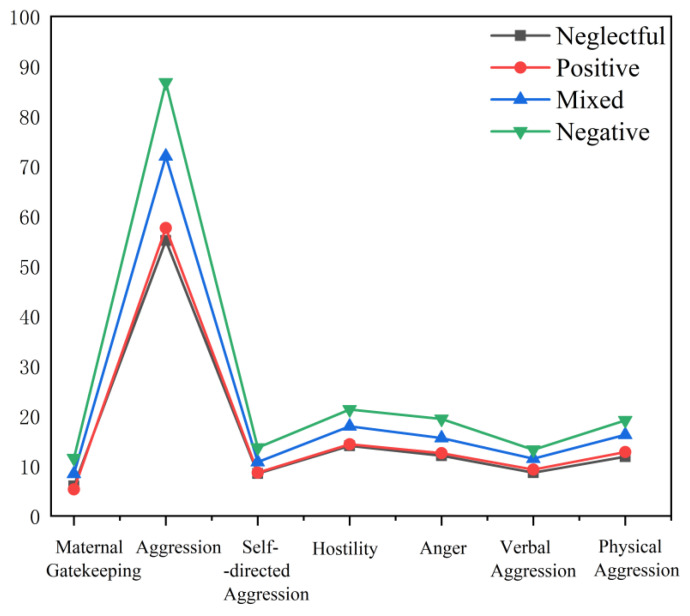
Maternal gatekeeping behavior and adolescent aggression.

To validate the effectiveness of the latent categories of parental rearing styles, analysis of variance (ANOVA) was conducted with the parental rearing style categories as the independent variable and maternal gatekeeping behavior, paternal parenting behavior, and paternal involvement (Figure 3) and adolescent aggression (Figure 4) as the dependent variables. The results revealed significant differences in the scores of maternal gatekeeping behavior (*F* (2,192) = 101.750, *p* < 0.001), parenting behaviors (*p_s_* < 0.001), paternal parenting involvement (*F* (2,192) = 101.750, *p* < 0.001), and adolescent aggression (*F* (2,192) = 101.750, *p* < 0.001) across different latent categories of parenting styles. The details of the post hoc comparison between the latent categories are displayed in the Supplemental Materials (Table S1).

### 3.4. Correlation Analysis

Among all subjects, Pearson correlation analysis showed that maternal gatekeeping behavior and parents’ negative parenting style were significantly positively correlated with adolescent aggression. In addition, both paternal positive parenting style and paternal parenting involvement were significantly negatively correlated with adolescent aggression. Moreover, maternal gatekeeping behavior was significantly positively correlated with paternal negative parenting style and negatively correlated with paternal positive parenting and paternal involvement (Table 3). Interestingly, maternal gatekeeping behavior was also positively correlated with maternal negative parenting style.

### 3.5. Moderated Mediation Model

In order to examine the relationship between the quality and quantity of paternal parenting and maternal gatekeeping behavior and adolescent aggression, this study proposed two moderated mediation models. After controlling the influences of gender, SES, and the education level of the parents, the results indicated significant effects for both models. However, when comparing the two models, Model 1 had an *R*^2^ of 0.17 for negative paternal parenting (*F* = 22.35, *p* < 0.0001), while Model 2 had an *R*^2^ of 0.14 for paternal involvement (*F* = 13.99, *p* < 0.0001), suggesting that Model 1 was superior to Model 2. According to the modulation effect, the negative impact of negative paternal parenting is more pronounced when fathers are more involved.

Further examination of Model 1 revealed that when the paternal negative parenting style was divided into refusal and overprotection, we found that paternal refusal showed a more pronounced influence (*R*^2^ = 0.18, *F* = 19.83, *p* < 0.0001) compared with overprotection (*R*^2^ = 0.11, *F* = 11.32, *p* < 0.0001). This suggests that mothers’ gatekeeping behavior is more closely related to fathers’ refusal than overprotection.

Importantly, all of the above results remained significant when analyzing the conventional families (Supplemental Materials Table S2, and Figures S1–S3) but not the non-conventional families.

## 4. Discussion

The present study advances our understanding of the potential dynamics between parental interactions and adolescent aggression. Through latent profile analysis, this paper categorizes parenting styles into four types: positive, negative, mixed, and neglectful. Each class demonstrates significant differences in maternal and paternal parenting behavior, as well as in adolescent aggression. The uniqueness of this study lies in the simultaneous consideration of the relationships between maternal gatekeeping behavior, the quality and quantity of paternal parenting, and adolescent aggressive behavior.

### 4.1. The Difference between Different Classifications of Pareting Styles

Our findings indicate that the parenting styles of fathers and mothers are relatively consistent, with both exhibiting similar levels of positive and negative behaviors within each type. This finding aligns with some other studies. To illustrate, in urban Chinese families with preschool-aged children, researchers found that the consistency between maternal and paternal parenting styles exceeded 70% [37]. Likewise, another study using data from a sample of 600 Flemish families raising an 8- to 10-year-old child also reported more similarities than dissimilarities in the parenting of both parents. Moreover, this similarity has been observed in other aspects of family life [38]. These similarities may reflect an assortative process in choosing a partner, meaning people tend to look for partners with similar characteristics [39]. In addition, according to the family systems theory, spouses tend to recognize and accept each other’s behaviors, making it easier to form a common parenting style, especially in a healthy marriage [40]. However, the consistency also extends to negative parenting. Negative parenting from one parent can induce stress on the other parent and the children, which, in turn, may trigger more negative parenting behaviors from the partner and lead to negative outcomes for the children. A study found significant cross-sectional associations between parenting stress, parenting behavior, and adolescent behavior problems [41].

As an essential component of parent-child interactions, parenting styles have a lasting impact on individuals’ lifelong development [42]. Research has repeatedly indicated that parenting styles may influence teenage aggression [43,44], with different types of parenting styles being differentially associated with adolescent aggressive behavior. In families characterized by a negative parenting style, for example, mothers exhibit the highest level of gatekeeping behavior, and fathers exhibit the highest level of negative parenting and the lowest involvement, while adolescents display the highest aggressive behavior. This is consistent with the findings of Luo Guiming [45]. These research findings were validated in the overall sample and in conventional families.

On the contrary, under the positive parenting style, mothers exhibit the least amount of gatekeeping behavior, fathers exhibit the lowest level of negative parenting and the highest involvement, and adolescents display the lowest aggressive behavior. Contrary to our expectation, adolescents from families with neglectful parenting also demonstrated the lowest aggressive behavior. The similarity between the two types of families suggests that it is the lowest level of negative parenting (including rejection and overprotection) that relates to the lowest adolescent aggression. Other studies support this interpretation [8,9,10].

### 4.2. The Relationship between Maternal Gatekeeping and Paternal Parenting and Adolescent Aggressive Behavior

In line with this interpretation, we found that negative parenting plays a significant role in adolescent aggression. More importantly, the significant mediation effect indicated that negative paternal parenting may be an unfavorable result of maternal gatekeeping [4]. The combination of maternal gatekeeping and negative paternal parenting contributes to adolescent aggression based on several reasons [46,47,48].

For one thing, gatekeeping behavior can undermine marital relationships. The family systems theory suggests that mothers’ restriction or negative judgment of paternal parenting might create marital conflicts. These conflicts are often regarded as detrimental environmental stimuli and direct triggers for various psychological, social, and emotional issues in adolescents. Moreover, parental conflicts can induce negative emotions, emotional insecurity, and lower self-efficacy in their children [48]. According to the general aggression model, both a toxic external environment and internal personal traits and emotional states are related to aggressive behavior. On the other hand, studies on the spillover hypothesis and the cascading transmission of parental conflicts indicate that adolescents’ aggressive behaviors might be imitations of their parents’ conflict resolution strategies. The social learning theory [49] suggests that through observation, imitation, and reinforcement, individuals develop patterns of conflict resolution learned from family interactions, which can then be transferred to peer interactions. Therefore, domestic conflicts and the solutions to parental conflicts may lead adolescents to perceive dominating, rather than cooperating, is a good way to survive conflicts. Alternatively, the aggressive behavior exhibited by adolescents may serve as a mechanism for diverting conflict between parents [50]. By manifesting aggressive conduct, it is possible that parents might overlook the conflict existing between themselves, consequently shifting their focus onto the child, which helps stabilize the family system.

Further analysis found that the mediating effect of negative parenting is mainly driven by paternal refusal, rather than overprotection, indicating that parental refusal, compared with overprotection, is more likely to lead to aggressive behavior. Reportedly, parents’ refusal poses long-term pressures of life that can hinder children’s self-control abilities [51], which is a core factor in triggering aggressive behavior [52], while being overprotective of children often leads to internalizing problems such as anxiety, depression, and withdrawal. This happens because overprotection conveys the incorrect belief that the outside world is uncontrollable and diminishes children’s self-awareness and emotional well-being [53]. There are at least two explanations for the closer relationship between paternal refusal and adolescent aggression. Firstly, children who experience parental refusal are more likely to experience frustration, which in turn can lead to the emergence of aggressive behavior according to the frustration–aggression theory [54,55]. Secondly, empirical evidence has demonstrated that children who experience parental refusal tend to have low self-esteem and poor self-adjustment, along with unstable emotions and negative worldviews [56], especially with refusal from fathers [57]. Given the close connection between low self-esteem/unstable emotions and externalizing problems [58], it is highly possible that aggression has been adopted as a tool to restore one’s self-esteem or emotion, especially in those adolescents who believe aggression is “cool” [59].

Strikingly, the significant moderated mediation effect implied that, under negative paternal parenting, the more the father was involved, the worse the adolescent aggression became. This is consistent with the findings of Zvara [26]. It is likely that an increase in negative parenting practices by fathers leads to more family conflict, causing greater frustration for adolescents and more significantly decreasing adolescent self-esteem and self-control. This, in turn, leads to an increase in aggressive behavior.

Although the hypothesized serial relationships between maternal gatekeeping behavior, paternal parenting behavior, and adolescent aggression are well supported, the observed results cannot rule out another possibility: that it is the father’s negative parenting that causes the maternal gatekeeping behavior. In other words, when mothers are dissatisfied with paternal involvement or parenting approaches, gatekeeping behavior may occur through either positive or negative feedback loops [60]. Hence, it is highly possible that paternal negativity may induce maternal gatekeeping behavior [61]. However, empirical studies have revealed that paternal characteristics, compared with maternal characteristics, have a relatively smaller predictive effect on maternal gatekeeping behavior [62]. For example, a perfectionistic mother may have higher expectations for the father’s parenting behavior, which may lead to low parenting confidence and limited experience for the father, making it difficult for him to meet the maternal standards and thus reinforcing the maternal gatekeeping behavior. Schoppe-Sullivan et al. [62] revealed that mothers with a belief in their superior parenting skills or those with high parenting efficacy are more likely to exert control over the father’s parenting style and involvement, leading to more gatekeeping behavior. Surprisingly, this study found that maternal gatekeeping behavior was significantly associated with mothers’ negative, rather than positive, parenting practices. Kulik and Tsoref [63] found that the more traditional a mother’s gender role consciousness, the more likely she is to believe that her partner is incapable of taking care of the child, leading to higher levels of gatekeeping behavior.

In a similar vein, empirical studies remind us that not only do parents’ caregiving behaviors influence children’s behaviors, but children also influence parents’ caregiving behaviors [64], a phenomenon known as the “evocative effect” [65]. For instance, one study found that infants’ negative emotions and lower regulatory abilities at 4–12 months predicted mothers’ negative parenting styles at 18 months [66]. Additionally, the bidirectional effects between children and their parents’ caregiving behaviors have been confirmed in longitudinal studies with older children [67,68]. Lee et al. discovered a bidirectional relationship between authoritarian parenting styles and children’s effortful control and anger/depression in Chinese families [67]. Therefore, it is very likely that adolescents’ aggressive behavior may elicit negative parenting behaviors as well, such as refusal and overprotection from parents, especially from those who take on more of the caregiving responsibilities [69]. Future studies using multiple waves of data would be better equipped to address such assumptions.

Of particular importance, our hypothesized model is significant in the whole sample and within the conventional sample but not within the non-conventional families. Hence, this likely indicates that the closer the family connections, the greater is the impact of parenting on adolescent aggressive behavior, and vice versa.

## 5. Limitations

This study highlights the complex interplay between maternal gatekeeping behavior, the quality and quantity of paternal parenting, and their potential impact on adolescent aggressive behavior, shedding light on how these familial dynamics contribute to a child’s behavioral development.

However, this study still has the following limitations. Firstly, employing self-reported methods for adolescents to measure perceived maternal gatekeeping behavior and perceived parental parenting styles may induce subjective bias. Future research could utilize a multi-informant approach to measure these variables. Secondly, the study adopts a cross-sectional design, which does not establish causal relationships between maternal gatekeeping behavior, paternal parenting behavior, and adolescent aggressive behavior. As mentioned above, it is possible that the relationships between maternal gatekeeping, paternal parenting, and adolescents’ behaviors could be bidirectional. Longitudinal studies using cross-lagged analysis may provide answers for this assumption. Thirdly, this study assumes a traditional family structure where the father is the primary breadwinner and the mother is the primary caregiver. However, the effects of this dynamic in atypical family structures, such as cases where the father is the primary caregiver and the mother is the breadwinner, remain to be investigated. After all, both parents are capable of engaging in gatekeeping behaviors [60]. Additionally, this study did not measure any factors that might influence mothers’ gatekeeping behavior, such as the mothers’ perfectionism, parenting efficacy, and marital quality. Although the results hold true for conventional families where the parents live together, marital quality can vary significantly within cohabitating families. Lastly, we missed the information on siblings, such as step-siblings and adoptive or foster siblings, which may sway the findings if multiple children are reporting on the same parents. Since we recruited adolescents in the seventh grade, only siblings of the same age or twins were likely to be involved simultaneously. Nevertheless, these caveats can be addressed in future research.

## 6. Conclusions

Grounded in the traditional Chinese cultural belief of “men as breadwinners and women as homemakers” and in line with the trend of societal development, this study explored the association between maternal gatekeeping behavior, paternal parenting quality and quantity, and adolescent aggressive behavior, taking into account the systemic and interactive features of modern families. In addition, this study also attempts to comprehensively explain the causes of adolescent aggressive behavior from the perspective of the family system.

Based on the co-parenting system theory, mothers should reduce criticism and blame towards the quality and quantity of paternal parenting while actively parenting children, help and support paternal parenting behaviors, and discuss child-rearing matters with fathers on an equal footing. Fathers should avoid excessive harshness and control in parenting children, offering more respect and autonomy to adolescents during their teenage years.

## Figures and Tables

**Table 1:** Demographic Variables and Difference Tests for Different Family Structures.

	Conventional Family Structure (N = 419)	Non-Conventional Family Structure (N = 64)	*t/X* ^2^	*p*
	M (SD)	Mean (SD)		
Age	12.66 (0.82)	12.66 (0.55)	0.03	0.98
Sex (M/F)	213/206	36/28	0.35	0.58
Maternal education	2.04 (0.78)	2.13 (0.98)	−0.79	0.43
Paternal education	2.17 (0.77)	2.17 (0.92)	0.06	0.95
SES	6.97(1.54)	6.30 ± 1.56	3.27	0.00
Maternal gate closing	6.15 (5.07)	9.16 (6.55)	−3.37	0.00
Maternal gate opening	13.08 (5.37)	8.25 (6.63)	5.31	0.00
Maternal warmth	21.24 (4.70)	18.95 (6.22)	2.70	0.01
Maternal refusal	9.23 (3.49)	10.41 (4.57)	−1.97	0.05
Maternal overprotection	17.11 (4.52)	17.33 (4.39)	−0.36	0.72
Paternal involvement	48.14 (18.33)	33.72 (22.69)	4.63	0.00
Paternal warmth	19.78 (5.05)	15.45 (6.54)	4.84	0.00
Paternal refusal	9.00 (3.61)	9.84 (4.96)	−1.29	0.20
Paternal overprotection	15.68 (4.19)	15.41 (4.11)	0.47	0.64
AQ_Total	60.90 (20.13)	66.71 (24.18)	−2.00	0.05

M: male; F: female; education levels were coded into 3 levels: 1 represents middle school education, 2 represents high school education, and 3 represents college and/or above; SES, subjective economic status; AQ, Buss–Perry aggression questionnaire.

**Table 2:** The fit indices of the latent profile analysis model.

Model	AIC	BIC	ABIC	Entropy	LMRLR (p)	BLRT (p)
**1**	23,085.336	23,143.885	23,099.450	----	----	----
**2**	22,263.725	22,355.731	22,285.905	0.917	0.0013	<0.0001
**3**	21,948.334	22,073.797	21,978.579	0.882	<0.0001	<0.0001
**4**	21,800.252	21,959.171	21,838.562	0.837	0.277	<0.0001

**Table 3:** The results of Pearson correlation analysis between variables.

	1	2	3	4	5	6	7	8
1 AQ_total	1							
2 Paternal warmth	−0.105 *	1						
3 Paternal refusal	0.398 **	−0.389 **	1					
4 Paternal overprotection	0.414 **	−0.151 **	0.650 **	1				
5 Paternal involvement	−0.113 *	0.695 **	−0.263 **	−0.040	1			
6 Maternal warmth	−0.069	0.730 **	−0.264 **	−0.162 **	0.443 **	1		
7 Maternal refusal	0.435 **	−0.292 **	0.708 **	0.525 **	−0.230 **	−0.402 **	1	
8 Maternal overprotection	0.412 **	−0.118 **	0.482 **	0.753 **	−0.029	−0.197 **	0.618 **	1
9 Maternal gate closing	0.274 **	−0.171 **	0.335 **	0.265 **	−0.185 **	−0.079	0.359 **	0.312 **

AQ, Buss–Perry aggression questionnaire; *, *p* < 0.05; ** *p* < 0.01.

## Data Availability

The raw data supporting the conclusions of this article will be made available by the authors, without undue reservation.

## Supplementary Materials

The following supporting information can be downloaded at: https://www.mdpi.com/article/10.3390/bs14070517/s1, Figure S1: The latent classes of parenting in conventional families; Figure S2: The differences in paternal parenting between different classes in conventional families; Figure S3: The differences in adolescents aggression between different classes in conventional families; Table S1: Differences in variables of interests between different latent categories; Table S2: Results of Pearson correlation analysis between variables in the conventional families.
