# Supplementary material for: Exploring the Relationship between Maternal Gatekeeping with Paternal Parenting and Adolescent Aggression
Source: Behav Sci (Basel). 2024 Jun 21;14(7):517. doi: 10.3390/bs14070517 (PMC11274204; doi:10.3390/bs14070517)
Supplement: Supplementary file 1 [file behavsci-14-00517-s001.zip › behavsci-2992620-supplementary.pdf]

Supplemental materials

Table S1. Differences in variables of interests between different latent categories

	Neglect（mean ± SD）	Positive（mean ± SD）	Mixed（mean ± SD）	Negative（mean ± SD）	*F*	*P*	*Post hoc* (LSD)
AQ_total	55.11±18.89	57.64±17.65	71.98±20.39	86.77±23.67	36.9	<0.001	1≈2
AQ_self	8.48±3.54	8.65±3.63	10.77±4.77	13.67±4.29	22.125	<0.001	1≈2
AQ_hostility	14.03±5.50	14.36±5.27	17.94±5.77	21.30±6.06	24.857	<0.001	1≈2
AQ_anger	12.05±5.26	12.56±5.28	15.58±5.96	19.42±6.46	22.377	<0.001	1≈2
AQ_verbal	8.67±3.32	9.28±3.22	11.47±3.80	13.25±5.05	23.568	<0.001	1≈2
AQ_physical	11.87±4.92	12.80±5.07	16.23±6.08	19.13±7.57	24.372	<0.001	1≈2
Paternal involvement	33.80±16.43	55.95±15.89	43.31±18.22	32.13±17.67	58.303	<0.001	1≈4
Paternal warmth	14.38±3.30	23.25±2.81	17.15±4.75	14.30±5.46	202.679	<0.001	1≈4
Paternal refusal	7.88±1.93	7.35±1.61	11.45±2.56	19.61±2.99	415.336	<0.001	
Paternal overprotect	13.47±2.59	14.31±2.77	18.8±3.53	24.09±3.32	166.12	<0.001	
Maternal warmth	16.95±3.62	24.44±2.46	18.28±4.66	17.64±5.68	154.621	<0.001	1≈4, 3≈4

Maternal refusal	8.32±2.16	7.62±1.73	12.45±3.15	17.27±3.92	228.055	<0.001	
Maternal overprotect	14.78±2.97	15.80±3.46	21.13±3.33	23.97±4.69	117.443	<0.001	
Gatekeeping	6.01±4.67	5.32/4.28	8.43±6.04	11.55±7.33	20.629	<0.001	1≈2
SES	6.76±1.61	7.292/1.47	6.340/1.40	5.97±1.55	14.324	<0.001	3≈4

AQ, Buss-Perry aggression questionnaire; SES, subjective economic status; Each variables of interests demonstrated significant differences between different latent categories. The last column listed post hoc comparison results. The group comparison yielded significant results except for the comparisons indicated in the last column. The "≈" means there is no significant differences between the specified two classes.

Table S2. Results of Pearson correlation analysis between variables in the conventional families

	1	2	3	4	5	6	7	8
1 AQ_total	1.00							
2 Paternal warmth	-0.098^{*}	1						
3 Paternal refusal	0.404^{**}	-0.384^{**}	1					
4 Paternal overprotect	0.447^{**}	-0.173^{**}	0.638^{**}	1				
5 Paternal involvement	-0.109^{*}	0.657^{**}	-0.278^{**}	-0.074	1			
6 Maternal warmth	-0.049	0.793^{**}	-0.281^{**}	-0.180^{**}	0.464^{**}	1		
7 Maternal refusal	0.425^{**}	-0.298^{**}	0.748^{**}	0.558^{**}	-0.246^{**}	-0.382^{**}	1	
8 Maternal overprotect	0.412^{**}	-0.123^{*}	0.483^{**}	0.778^{**}	-0.047	-0.198^{**}	0.625^{**}	1
9 Maternal gatekeeping	0.296^{**}	-0.103^{*}	0.284^{**}	0.280^{**}	-0.132^{**}	-0.065	0.349^{**}	0.336^{**}

AQ: buss-perry aggression questionnaire; *, $p < 0.05$; **, $p < 0.01$.

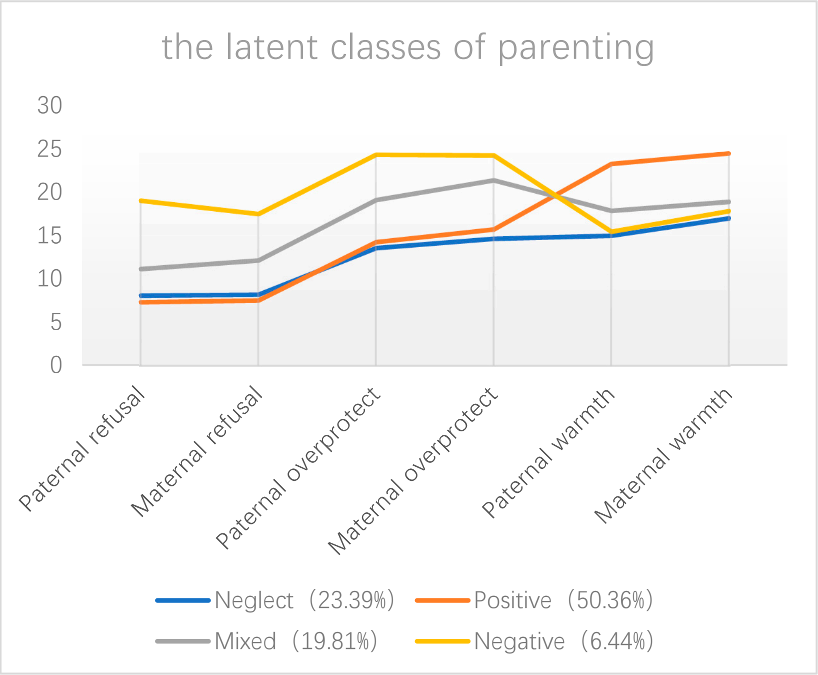

Figure S1. The latent classes of parenting in conventional families

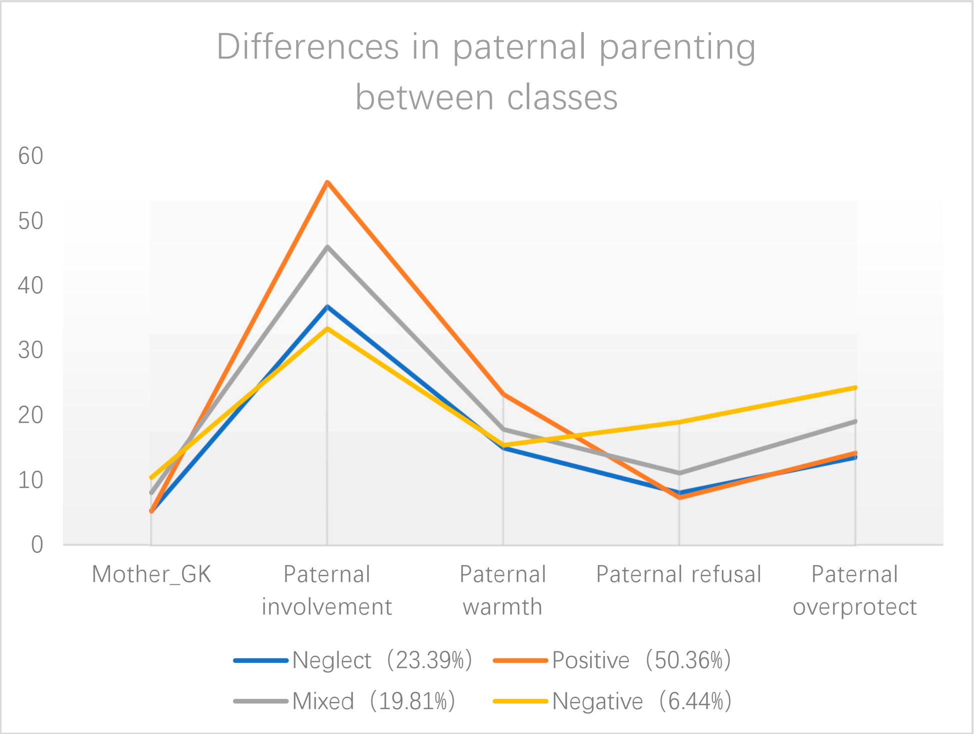

GK: gatekeeping behavior;

Figure S2. The differences in paternal parenting between different classes in conventional families

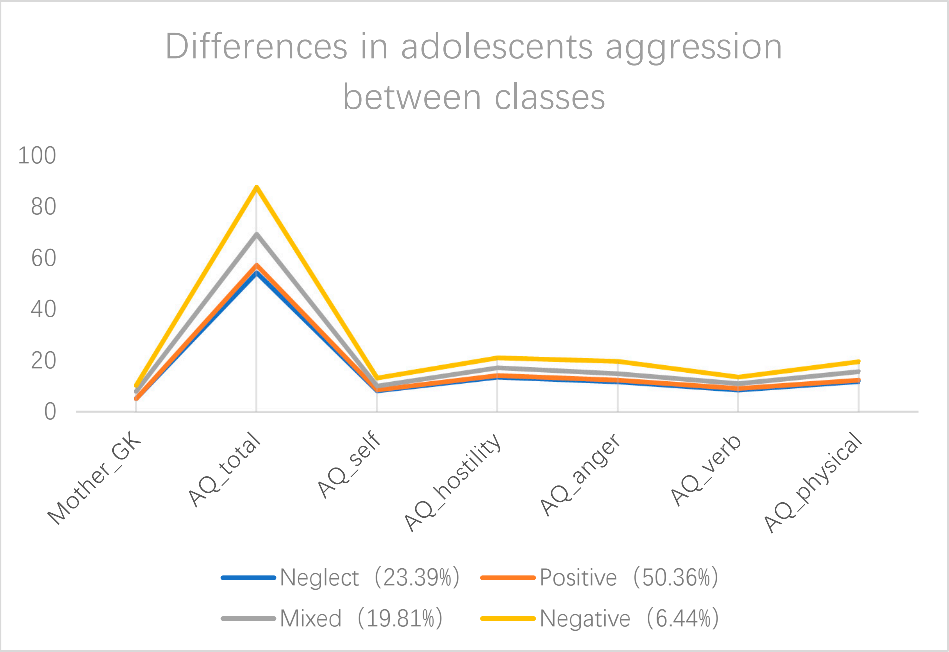

GK: gatekeeping behavior; AQ: Buss-perry aggression;

Figure S3. The differences in adolescents aggression between different classes in conventional families
